# Alterations in PGC1α expression levels are involved in colorectal cancer risk: a qualitative systematic review

**DOI:** 10.1186/s12885-017-3725-3

**Published:** 2017-11-09

**Authors:** Jéssica Alonso-Molero, Carmen González-Donquiles, Tania Fernández-Villa, Fernanda de Souza-Teixeira, Laura Vilorio-Marqués, Antonio J. Molina, Vicente Martín

**Affiliations:** 1 Grupo de Investigación en Interacciones Gen-Ambiente y Salud, Universidad de León, León, Spain; 2 Universidad de Cantabria, Santander, Spain; 3 Superior Physical Education School, Federal University of Pelotas, Pelotas, Brazil; 4 CIBER Epidemiología y Salud Pública (CIBERESP), Madrid, Spain; 5 Departamento Medicina Preventiva y Salud Pública, Facultad Ciencias de la Salud, Campus Vegazana, s/n. León, C.P.: 24071 Castilla y León, Spain

**Keywords:** PGC1α or PPARGC1α, Colorectal cancer (CRC), Signaling or metabolic pathways, Molecular mechanism

## Abstract

**Background:**

Colorectal cancer (CRC) is a major global public health problem and the second leading cause of cancer-related death. Mitochondrial dysfunction has long been suspected to be involved in this type of tumorigenesis, as supported by an accumulating body of research evidence. However, little is known about how mitochondrial alterations contribute to tumorigenesis. Mitochondrial biogenesis is a fundamental cellular process required to maintain functional mitochondria and as an adaptive mechanism in response to changing energy requirements. Mitochondrial biogenesis is regulated by peroxisome proliferator-activated receptor gamma coactivator 1-α (PPARGC1A or PGC1α). In this paper, we report a systematic review to summarize current evidence on the role of PGC1α in the initiation and progression of CRC. The aim is to provide a basis for more comprehensive research.

**Methods:**

The literature search, data extraction and quality assessment were performed according to the document Guidance on the Conduct of Narrative Synthesis in Systematic Reviews and the PRISMA declaration.

**Results:**

The studies included in this review aimed to evaluate whether increased or decreased PGC1α expression affects the development of CRC. Each article proposes a possible molecular mechanism of action and we create two concept maps.

**Conclusion:**

Our systematic review indicates that altered expression of PGC1α modifies CRC risk. Most studies showed that overexpression of this gene increases CRC risk, while some studies indicated that lower than normal expression levels could increase CRC risk. Thus, various authors propose PGC1α as a good candidate molecular target for cancer therapy. Reducing expression of this gene could help to reduce risk or progression of CRC**.**

**Electronic supplementary material:**

The online version of this article (10.1186/s12885-017-3725-3) contains supplementary material, which is available to authorized users.

## Background

Colorectal cancer (CRC) is a major global public health problem and the second leading cause of cancer-related death. It is the third most commonly diagnosed cancer in men and the second in women [1]**.** CRC is the seventh and fourth most common cause of death and loss of life expectancy in Western Europe, respectively, and is associated with an elevated consumption of resources [2, 3].

Mitochondrial dysfunction has long been suspected to be involved in this type of tumorigenesis, as supported by an accumulating body of research evidence. However, little is known about how mitochondrial alterations contribute to tumorigenesis [4–7]. Mitochondrial biogenesis is a fundamental cellular process required to maintain functional mitochondria and as an adaptive mechanism in response to changing energy requirements [8]. Both endogenous and exogenous factors, as well as numerous signaling pathways and gene expression patterns, converge upon the mitochondrial biogenesis process to coordinate the energy needs of cells, tissues and the entire organism [4, 8, 9].

The master regulator of mitochondrial biogenesis is peroxisome proliferator-activated receptor gamma coactivator 1-α (PPARGC1A or PGC1α), because it controls production of mitochondrial proteins [10]. This gene is a transcriptional coactivator of the PGC-1 (peroxisome proliferator-activated receptor gamma coactivator 1) gene family, which has three known members, PGC1α, PGC1β and PRC (PGC-1 related coactivator). PGC1α and PGC1β are expressed in tissues with high energy demand, while PRC is expressed ubiquitously [10, 11]. While all three members of this family are potent regulators of mitochondrial function and biogenesis, PGC1α is the most widely studied, and the other two are less well characterized [8, 11–13]. However, [11, 14]**.**

PGC1α acts as a master regulator of energy metabolism and mitochondrial biogenesis by integrating and coordinating the activity of other transcription factors, such as Nuclear respiratory factor 1, Nuclear factor 2, PPARα (peroxisome proliferator-activated receptors α) and Mitochondrial transcription factor A [15]. Various endogenous and exogenous factors also regulate mitochondrial biogenesis through this gene [4, 9]. In addition, expression levels of PGC1α appear to be directly related to mitochondrial biogenesis activity. As a multi-response factor, many agents and events regulate PGC1α expression via multiple intracellular mediators [16].

Several mutations in nuclear and mitochondrial genes encoding for mitochondrial components have been reported to be associated with increased cancer risk [17], and mitochondrial loss is known to precede the development of dysplasia [6]. We believe that there is a clear relationship between CRC and PGC1α; however, the role of mitochondria and PGC1α in CRC is poorly understood at present.

Several studies suggest that PGC1α and related genes can regulate different pathways, such as mitochondrial biogenesis, antioxidant systems, reactive oxygen species, de novo lipid synthesis, and glycolysis, there by playing a role in risk for and development of CRC (See discussion) [10, 11]. Although there is no widely accepted mechanism to explain how PGC1α is involved in human CRC, it is essential to understand this mechanism in order to reduce CRC risk, as well as the development of novel therapeutic tools to treat tumors and to support measures to reduce CRC risk.

In this paper, we report a systematic review to summarize current evidence on the role of PGC1α in the initiation and progression of CRC. Since there is a limited evidence on this specific question, we used a broad, inclusive search strategy, with the aim of providing a basis for more comprehensive investigation.

## Methods

### Search strategy

The literature search, data extraction and quality assessment were performed according to the document Guidance on the Conduct of Narrative Synthesis in Systematic Reviews [18] and the PRISMA declaration [19]. This search was made between June and September of 2016.

We conducted a web-based search of 8 databases (Cochrane library, PubMed, Scopus, Web of Science, PsycINFO, Scielo, PLoS One and PubMedCentral (PMC)), using the following search terms: "Colorectal OR Colon OR Rectum OR Rectal" and "Cancer OR Carcinoma OR Tumor OR Tumour OR Neoplasm OR Cancer Cells" and "Mitochondrial Biogenesis OR Mitochondrial dysfunction OR Mitochondria OR Mitochondrion" and “Warburg effect” and "OXPHOS OR Oxidative phosphorylation OR Anaerobic glycolysis" and "PGC1A OR PPARGC1A OR Peroxisome Proliferator-activated Receptor gamma coactivator 1 alpha". The search was restricted to English language articles. We used this quite broad search strategy because of the paucity of research on this specific issue.

### Selection process

The list of articles obtained by this search was manually screened to identify relevant articles. We first read the titles and removed irrelevant articles, and then read the abstracts to eliminate those not directly related to the objective of this review. We imported the resulting set of articles into a reference management program (Endnote), which allowed us to detect duplicate articles. Finally, we read the full text and decided if the article should be included in this review according to the exclusion criteria described below.

### Study selection

We applied the following exclusion criteria during each stage of the selection process mentioned above:

1) Title – We eliminated articles that did not deal with cancer or inflammatory bowel diseases (e.g. Ulcerative Colitis or Crohn’s disease, see below). We also excluded articles dealing with genes other than PGC1α or PGC1β (there is evidence that both have a similar role in the organism).

2) Abstract – We eliminated articles that did not directly deal with CRC or inflammatory bowel diseases that ultimately develop into CRC, or did not deal with some isoform of PGC1 (α or β), or with related pathways.

3) Full-text – i) We included articles on basic research such as with cell lines or animals. For human studies, we included both basic research, and population-based observational studies. ii) We included articles on colorectal cancer, as well as those on other diseases, such as ulcerative colitis, where these ultimately deal with CRC. We did not consider demographic factors during study selection because we considered a broad range of study types, including basic research.

4) The online search was replicated independently by two reviewers, who reviewed and filtered the titles, and created a draft list of titles. This list of articles to be included was agreed upon by three people (with the first author) based on the exclusion/inclusion criteria. The reviewers then independently reviewed the full text of the articles according to the inclusion criteria. Discrepancies were discussed and resolved in collaboration with the principal investigator.

### Preliminary synthesis

We used tabulation and visual representations of data to reduce studies to their key characteristics, which could be important for understanding the objective of this review.

### Relationship between papers

#### Evaluating heterogeneity

In general terms, this technique focused on the characteristics of the various studies and their potential relationship with the findings. The following characteristics were assessed:

- Cells lines (human or animals = 1)
- Colorectal cancer (Inflammatory bowel diseases as previous disease = 1)
- PGC1α or PGC1β expression
- Relationship with other genes
- reactive oxygen species
- Mitochondrial biogenesis
- Chemotherapy

These characteristics were scored as 1 if they were present and 0 if absent. We took the sum of these values to obtain a picture of the heterogeneity of the articles in this review. Using these values, we created a comparison graph to evaluate the shared features.

#### Idea webbing and concept mapping [18]

Idea webbing is a method for conceptualizing and exploring connections among the findings reported by the studies included in the review (data not shown). Using idea webbing we obtained a concept map, a visual picture linking multiple pieces of evidence across several studies. The aim was to construct a model of key concepts related to PGC1α and PGC1β, and to represent the relationships between these and the development of CRC.

#### Checking the synthesis with authors of primary studies

We compared our results to those of other systematic reviews to support our ideas.

## Results

### Literature search results

**Fig. 1:**
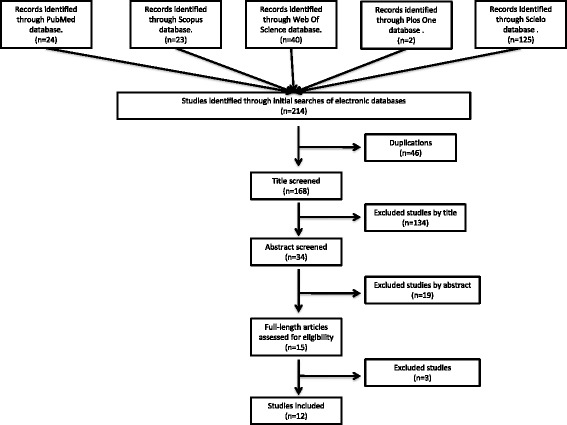
Summary of article selection process

Using the search terms described in the Methods section, but excluding the Boolean operators, we identified 7688 manuscripts from our search of PubMed, Scopus, Web Of Science (WOS), PsycInfo, Cochrane, Scielo and PLoSOne (no results were returned by PsycInfo or Cochrane). When the Boolean operators were included, 214 articles were returned (Fig. 1). We retained and analyzed 34 papers that met the eligibility criteria described in the Methods section. Of these 34 abstracts, 15 full-text articles were retrieved for detailed evaluation and 12 studies were included in the final analysis. Figure 1 illustrates the article screening and selection process.

Eighteen articles were excluded for the following reasons: i) Article mentions other genes related to PGC1α, but does not deal with PGC1α itself. ii) Article mentions PGC1α, but does not deal with CRC. iii) Article mentions PGC1α and its relationship with cancer, but does not deal with CRC. iv) Article mentions PGC1α and its relationship with inflammatory bowel diseases, but does not deal with CRC. v) Article mentions PGC1α and colorectal cancer but not define a relationship between them. A full summary of these data is shown in Additional file 1: Table S1 online.

### Preliminary synthesis

We chose tabulation to visually represent the data, with the aim of reducing studies to the key characteristics that could be important for understanding the relationship between PGC1α and CRC. The key message observed in these articles was that patterns of altered PGC1α expression affect risk and development of CRC via various molecular mechanisms, including mitochondrial biogenesis, antioxidant systems, reactive oxygen species, de novo lipid synthesis, glycolysis and alterations in the expression of other genes. Most of this information was obtained from basic research, such as cell culture, since there is little information about this field. These data are summarized in Table 1, and full details are provided in Additional file 1: Table S2 online. In addition, Table 2 summarizes the original material used in each of these articles.

**Table 1:** Summary of the key characteristics of the selected studies

Title	First Author	Year	Primary results	Conclusion
Overexpression of PGC1-alpha enhances cell proliferation and tumorigenesis of HEK293 cells through the upregulation of Sp1 and Acyl-CoA binding protein.	Sung-Won Shin	2014	1) PGC-1α accelerates proliferation of HEK293 and CT-26 cells. 2) Knockdown of PGC1-α expression results in decreased cell proliferation of human colorectal cancer cells. 3) PGC-1α promotes the oncogenic potential of HEK293 cells. 4) PGC-1αoverexpressing HEK293 cells have decreased sensitivity to oxidative stress.	PGC-1α overexpression upregulates proliferation of HEK293 and CT26 cells. In addition, this expression correlates with enhanced tumorigesis. Moreover, PGC-1α siRNA transfection resulted in decreased cell proliferation. Further studies to clarify the molecular interactions are needed.
PGC-1β promotes enterocyte lifespan and tumorigenesis in the intestine	Elena Bellafante	2014	1) PGC-1β Is Highly Expressed in the Intestinal Epithelium and Modulates Intestinal Morphology. 2) Intestinal PGC-1β Overexpression Enhances Antioxidant Defense. 3) Intestinal PGC-1β Overexpression Promotes Intestinal Carcinogenesis.	PGC-1β seems to act as an adaptive self-point regulator, capable of providing a balance between mitochondrial activity and production of increased reactive oxygen species.
Mitochondria and Tumor Progression in Ulcerative Colitis	Cigdem Himmetoglu Ussakli	2013	1) Comparison of COX in No dysplastic Biopsies of UC Progressors and Nonprogressors show that tumor development could be due to previous lower COX levels. 2) Cox levels increase after the tumor development (Bimodal pattern). Mitochondria follow the same pattern. 3) PGC1α may drive of the mitochondrial changes observed.	1) At the biomarker level, COX loss precedes tumor progression in UC. 2) At the biological level, the loss of COX represents a reduction in the number of mitochondria in preneoplasia, which is restored in cancers. It appears to be driven by PGC1α.
PGC1α promotes tumor growth by inducing gene expression programs supporting lipogenesis.	Kavita Bhalla	2011	1) Loss of PGC1 protects against both colon and liver tumorigenesis. 2) Overexpression PGC1α promotes tumor growth in vivo. 3) PGC1α mediated induction of fatty acid synthesis promotes tumor growth.	1) Novel role for PGC1α in promoting carcinogenesis and tumor growth. 2) PGC1α coordinates the induction of a gene expression program that facilitates the conversion of glucose to fatty acids. 3) PGC1α is a potential therapeutic target for chemoprevention.
Bax is necessary for PGC1α pro-apoptotic effect in colorectal cancer cells	Ilenia D’Errico	2011	1) PGC1α induces Bax activation. 2) PGC1α increases mitochondrial activity. 3) PGC1α induces apoptosis in the presence of Bax, but not without Bax. 4) PGC1α inhibits tumor growth in presence of Bax.	1) In the presence of Bax, the PGC1α-induced accumulation of reactive oxygen species is one of the main apoptosis-driving factors in CRC cells. 2) PGC1α is able to induce Bax activation and translocation to mitochondria, thus leading to apoptotic cascade.
PGC-1α/β upregulation is associated with improved oxidative phosphorylation in cells harboring nonsense mtDNA mutations	Sarika Srivastava	2007	1) PGC-1α and PGC-1β are markedly upregulated in V425. 2) Overexpression of PGC-1α and PGC-1β transcriptional coactivators stimulates mitochondrial respiration, at least in osteosarcoma cybrids. 3) Overexpression of PGC-1α stimulates complex IV activity. 4) Overexpression of PGC-1α/β transcriptional coactivators can stimulate respiration in oxidative phosphorylation-deficient cells.	1) In V425 cells, the Ca2 + −dependent signaling events are active for relatively longer periods, which in turn might activate the nuclear genes (including PGC-1α/β) involved in tumor invasion and metastasis. 2) Overexpression of PGC-1α/β can stimulate respiration in oxidative phosphorylation deficient cells. 3) This pathway could be explored as a therapeutic approach for the treatment of human mitochondrial diseases.
Validation of the Use of DNA Pools and Primer Extension in Association Studies of Sporadic Colorectal Cancer for Selection of Candidate SNPs	Mette Gaustadnes	2006	Results were analyzed using the χ2 test with a level of significance α = 0.05. Five SNPs were found. The SNP analysis of the (*604517)3’utr96516 was not reproducible, but it was always statistically significant.	The results of this article allow us to conclude that the difference between cases and controls would be statistically significant for n = 600 cases and *n* = 600 controls.
SIRT1/PGC1a-Dependent Increase in Oxidative Phosphorylation Supports Chemotherapy Resistance of Colon Cancer	Thomas T.Vellinga	2015	1) Chemotherapy induces SIRT1 to promote oxidative energy metabolism. This gene controls mitochondrial biogenesis by deacetylation and activation of PGC1α. 2) SIRT1 and PGC1α protect colon cancer cells against chemotherapy.	Colorectal tumors shift their energy metabolism when challenged with chemotherapy. Chemotherapy induces oxidative phosphorylation in colon cancer cells via the SIRT1/PGC1a axis to help them survive treatment.
AMPK Promotes Aberrant PGC1β Expression To Support Human Colon Tumor Cell Survival	Kurt W. Fisher	2015	PGC1α is not detected in HCT116 cell line. 1) PGC1β and ERRα are key downstream effectors of K-Ras, KSR1, and AMPK1. 2) Both AMPK 1 and K-Ras depletion decreased the protein levels of PGC1β. 3) PGC1β and ERRα are overexpressed in colon cancer and are required for colon cancer survival both in vivo and in vitro.	The aberrant expression of PGC1β and ERRα that persists in additional tumors with oncogenic Ras alleles will reveal the importance of these transcriptional regulators in creating tumor cells and promoting their survival. This may represent a new therapeutic target.
Peroxisome proliferator-activated receptor-γcoactivator 1-α (PGC1α) is a metabolic regulator of intestinal epithelial cell fate	Ilenia D’Errico	2011	1) Expression level of PGC1α in the intestine is higher in differentiated enterocytes than in the proliferative compartment at the bottom of the crypts, where it has only a scattered expression. 2) PGC1α Induces Mitochondrial Proliferation and Activation in Human Intestinal Cancer Cells. 3) PGC1α induces tissue-specific accumulation of reactive oxygen species and apoptosis. 4) PGC1α Stimulates Intestinal Mitochondrial Biogenesis and Respiration in vivo, and suppresses Colorectal Carcinogenesis	1) PGC1α expression levels could influence intestinal epithelial cell fate by inducing mitochondrial-related metabolic modifications that induce apoptosis. 2) PGC1α overexpression stimulates mitochondrial biogenesis, metabolic activities and accumulation of reactive oxygen species. 3) In tissues with high aerobic energy demand, PGC1α preserves reactive oxygen species‘homeostasis; In normal intestine, PGC1α cannot induce reactive oxygen species scavenging systems.
Peroxisome Proliferator-Activated Receptor Coactivator-1alpha Enhances Antiproliferative Activity of 5′-Deoxy-5-Fluorouridinein Cancer Cells through Induction of Uridine Phosphorylase	Xingxing Kong	2009	1) PGC-1 Induces the Expression of UPase in Breast and Colon Cancer Cells. 2) PGC1α-Dependent Induction of UPase Gene in Cancer Cells Is Mediated by ERRα. 3) Overexpression of PGC-1 Sensitizes Cancer Cells to 5 -DFUR.	1) PGC-1αseems to be a regulator of UPase gene transcription, whose effect is mediated by ERRα. 2) PGC1α has an effect on the absence of ERRα, suggesting the involvement of other regulatory factors. 3) In tumor cells, UPase catalyzes the transformation of 5 -DFUR to 5-FU, which inhibits their proliferation. In this way, PGC1α enhances the cell’s sensitivity to the treatment.
Peroxisome proliferator-activated receptors (PPARs) and associated transcription factors in colon cancer: reduced expression of PPARg-coactivator 1 (PGC-1)	Jonas Feilchenfeldt	2004	1) RXRα expression in tumors is similar relative to normal mucosa. 2) PGC-1 expression in the tumors was significantly decreased relative to normal mucosa.	1) PPARβ/δ may repress PPARα and PPARϒ target gene expression. 2) Reduced coactivator levels ofPGC-1 are compatible with reduced transcriptional activity of PPARϒ and hence reduced tumor suppressor activity. 3) Transcriptional activity of PPARϒ may not only be decreased by mutation and increased levels of the transcriptional repressor PPARβ/δ but also by downregulation of coactivator PGC-1 of PPARϒ.

**Table 2:** Cell lines and other materials, and characteristics of articles considered in this review

Title	Main Material	Characteristics
Overexpression of PGC-1α enhances cell proliferation and tumorigenesis of HEK293 cells through the upregulation of Sp1 and Acyl-CoA binding protein	HT-29 (Cell line)	i) Human.
		ii) Epithelial
	SNU-C4 (Cell line)	i) Human
	CT-26 (Cell line)	i) Mouse.
		ii) Epithelial
PGC-1β promotes enterocyte lifespan and tumorigenesis in the intestine	iPGC1b mouse model with human PGC1b	i) Transgenic mouse
	iPGC1b knockout mice	
	iPGC1b Apc Min/+ Mice	
Mitochondria and Tumor Progression in Ulcerative Colitis	Ulcerative Colitis progressor (Human)	i) Human
	Ulcerative Colitis non progressor (Human)	
PGC1α promotes tumor growth by inducing gene expression programs supporting lipogenesis.	PGC1a knockout mice	i) Transgenic mouse
	PGC1a +/+ mice	
	SCID mice (HT29)	i) Inoculated mouse
	HT29 (Cell line)	i) Human.
		ii) Epithelial
	Colo205 (Cell line)	i) Human.
		ii) Epithelial
Bax is necessary for PGC1α pro-apoptotic effect in colorectal cancer cells	HCT116 (Cell line) Nude mice (subcutaneously injected both cells)	i) Human ii) Epithelial i) Inoculated mouse
PGC-1α/β upregulation is associated with improved oxidative phosphorylation in cells harboring nonsense mtDNA mutations	VACO425 (Cell line)	i) Human
	VACO429 (Cell line)	i) Human
Validation of the Use of DNA Pools and Primer Extension in Association Studies of Sporadic Colorectal Cancer for Selection of Candidate SNPs	Two pools of genomic DNA (patients with sporadic CRC + controls)	i) Human
SIRT1/PGC1a-Dependent Increase in Oxidative Phosphorylation Supports Chemotherapy Resistance of Colon Cancer	Colonosphere cultures shSIRT1 (Human colorectal tumor specimens)	i) Human colonosphere cultures
	Colonosphere cultures shPGC1a (Human colorectal tumor specimens)	
AMPK Promotes Aberrant PGC1b Expression To Support Human Colon Tumor Cell Survival	HCT116 (Cell line)	i) Human.
		ii) Epithelial
	Inmunodeficient mice (HCT116 cells grafted into mice)	i) Inoculated mouse
Peroxisome proliferator-activated receptor-γ coactivator 1-α (PGC1α) is a metabolic regulator of intestinal epithelial cell fate	HT29 (Cell line)	i) Human.
		ii) Epithelial
	HCT116 (Cell line)	i) Human.
		ii) Epithelial
	HT29p0 (Cell line: Completely lacks mitochondrial DNA)	i) Human.
		ii) Epithelial
	Xenograft mice using HT29 cells	i) Inoculated mouse
	iPGC1a transgenic mice	i) Transgenic mouse
	PGC1a +/+ mice and PGC1−/− mice	
Peroxisome Proliferator-Activated Receptor gamma Coactivator-1 Enhances Antiproliferative Activity of 5 -Deoxy-5-Fluorouridine in Cancer Cells through Induction of Uridine Phosphorylase	Colo320 (Cell line)	i) Human.
		ii) Undifferentiated
	HCT116 (Cell line)	i) Human.
		ii) Epithelial
Peroxisome proliferator-activated receptors (PPARs) and associated transcription factors in colon cancer: reduced expression of PPARg-coactivator 1 (PGC-1)	Colorectal cancers from patients (Human)	i) Human

### Relationships between papers

#### Heterogeneity

**Fig. 2:**
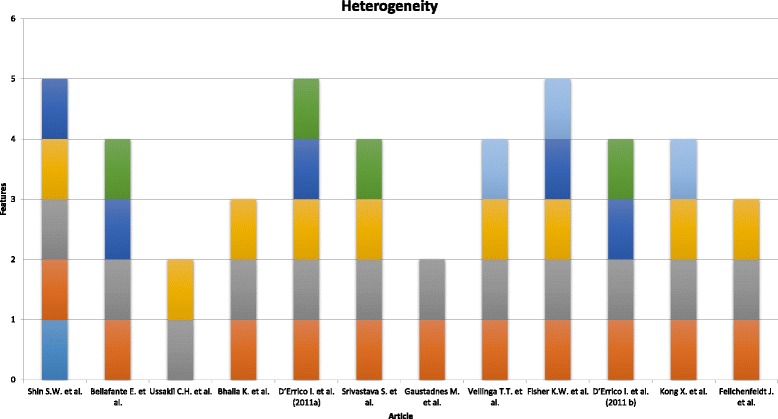
Heterogeneity assessment

We analyzed differences in relevant characteristics between the selected articles studied using the graph shown in Fig. 2.

We assessed the qualitative outcomes of the 12 studies in terms of 6 features: study of CRC; assessment of PGC1α or PGC1β (which should be present in all papers); relationship between PGC1α and another gene; test for presence of reactive oxidative species; study of mitochondrial biogenesis; study of chemotherapy. Four papers (30.8%) had a maximum score of five points, and another 4 articles (30.8%) had a score of four points. One study (7.6%) had three points, two (15.4%) had two points, and another two (15.4%) had six points. These proportions show that the studies were sufficiently homogeneous to perform a systematic narrative review.

#### Concept mapping

**Fig. 3:**
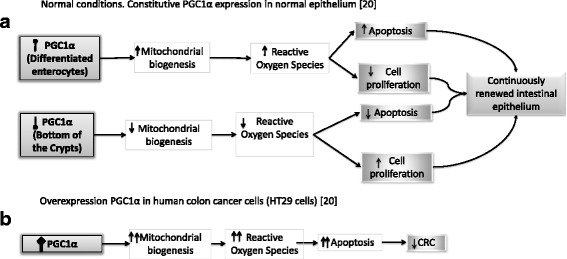
Concept map based on D’Errico et al. Ref [20]

Based on the information in Table 1, we performed idea webbing for each article, from which we obtained two concept maps. The first concept map is based on D’Errico et al. [20], whose aim was to show that PGC1α is highly expressed on the surface of the intestinal epithelium but is poorly expressed in the crypts, and is also reduced in intestinal tumors. D’Errico et al. analyzed the expression and function of PGC1α along the crypt-to-villus axis under normal conditions, and observed that PGC1α is poorly expressed in the proliferative compartment at the bottom of the crypts, but, conversely, is highly expressed at the villus tips, promoting mitochondria-mediated apoptosis via the accumulation of reactive oxygen species (Fig. 3a).

These authors also identified that overexpression of PGC1α in human colon cancer cells (HT29) activates metabolic changes such as mitochondrial activation, which produce a proapoptotic effect via reactive species oxygen accumulation (Fig. 3b). Thus, they conclude that PGC1α is a metabolic regulator of intestinal cell fate and protects against tumorigenesis.

**Fig. 4:**
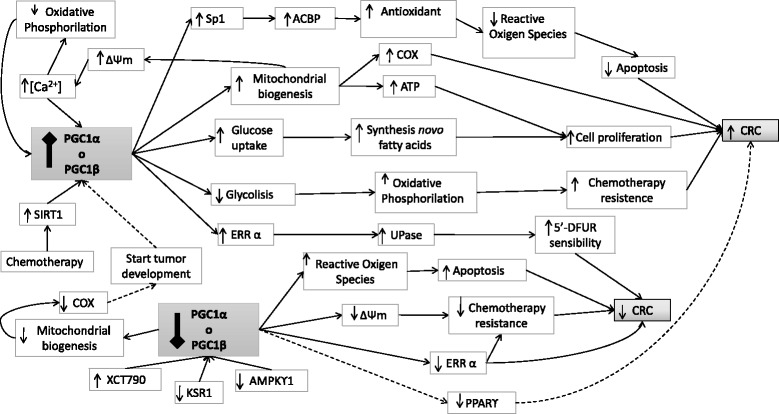
General concept map

The second concept map highlights patterns of altered gene expression in the development of colorectal cancer. Most papers included in this concept map suggest that PGC1α expression is increased during the development of CRC, while reduced PGC1α expression reduces risk and progression of this disease. Nonetheless, some studies have reported the opposite, that reduced PGC1α expression levels can also increase cancer risk (Fig. 4).

Figure 4 shows various possible mechanisms of action of PGC1α in relation to CRC development, and suggests four different mechanisms via which this gene can become overexpressed. First, high expression of PGC1α seems to increase Antioxidant systems, which reduces the number of reactive oxygen species, resulting in inhibition of apoptosis. Second, overexpression of PGC1α induces the mitochondrial biogenesis pathway, increasing cellular growth (proliferation). Third, overexpression of PGC1α induces glucose uptake, increasing cell proliferation. Fourth, high levels of PGC1α could reduce glycolysis and increase oxidative phosphorylation, which increases cells’ resistance to chemotherapy. In the map, we can observe certain factors that trigger overexpression of PGC1α, such as the mitochondrial biogenesis pathway (via positive feedback), or chemotherapy.

In contrast, cancer development can also be promoted by silencing or downregulation of PGC1α, also via two possible mechanisms. First, low levels of PGC1α hinder the correct function of the mitochondrial biogenesis pathway, which promotes CRC growth. Second, low levels of PGC1α reduce PPAR-ϒ, which promotes CRC growth via an unknown process.

Figure 4 highlights some mechanisms that reduce CRC risk or development. In this case, reduction of PGC1α levels is due to inhibition of KSR1 and AMPK-ϒ1 or XCT790 chemotherapy, via three possible mechanisms. First, lack of PGC1α increases reactive oxygen species levels, which activates apoptosis. Second, reduced mitochondrial membrane potential (ΔΨm) reduces resistance to chemotherapy. Third, low expression of PGC1α seems to reduce ERRα expression, reducing CRC. In contrast, other mechanisms support the idea that PGC1α overexpression increases ERRα expression, which may increase chemotherapy sensitivity.

These contrasting results could be due to the different starting material (e.g. cell lines, mice, etc.) in these studies. In Table 2 we summarize the material used in each article with the objective of trying to understand the cause of these differences. Note that we only indicate the main starting material, from which the authors obtained the principal results.

#### Checking the synthesis with authors of primary studies

We were unable to compare our results to those of other systematic reviews because no other reviews have dealt with this topic, as far as we are aware.

## Discussion

The central question of this review is clear: summarize current evidence on the role of PGC1α in the initiation and progression of CRC. We tested the effect of different PGC1α expression levels during the development of colorectal cancer. There is little prior evidence on this question, so only a small number of studies have been included in this review.

The studies included in this review aimed to evaluate whether an increase or decrease in PGC1α expression levels affects the development of CRC. Most studies (61.5%) were carried out using cell lines [10, 11, 20, 21, 23–25]: in four studies, cells were injected subcutaneously into the flanks of mice [20, 21, 23, 24]**,** and two studies evaluated expression levels in mice in vivo [14, 21]. Other studies were carried out in samples of patients with disease [6, 13, 26, 27], with similar results to the in vitro studies.

Each article proposes a possible molecular mechanism of action. First, we try to understand the normal mechanism of PGC1α action proposed by Ilenia D’Errico et al. [20], which is derived from work on cell cultures and two groups of transgenic mice: iPGC1α transgenic mice and iPGC1α Apc^Min/+^ mice (more information in Table 2). The authors conclude that PGC1α is a metabolic regulator of intestinal cell fate. In addition, PGC1α seems to have different functions in tissues with high aerobic energy metabolism, such as at the bottom of the crypts, where PGC1α expression increases production of antioxidant enzymes that protect cells from reactive oxygen species. In contrast, at the top of the villi, where aerobic energy metabolism is low, PGC1α expression increases mitochondrial biogenesis, resulting in greater accumulation of reactive oxygen species than at the bottom of the crypts, which induces apoptosis [20]. In this way, there is a balance between cell proliferation and apoptosis under normal conditions.

Other mechanisms have been proposed on the basis of various studies of PGC1α overexpression under non-normal conditions, mainly in cell culture. One mechanism supports the idea that overexpression of PGC1α results in high levels of Sp1 (specificity protein 1), which is then thought to enhance expression of ACBP (Acyl-CoA-binding protein) via ACBP’s Sp1 binding site. ACBP upregulation increases cell proliferation and decreases sensitivity to H2O2-induced apoptosis [10, 14]. A second mechanism proposes that PGC1α is a key metabolic regulator of several aspects of glucose metabolism. PGC1α is thought to coordinate gene expression in metabolic pathways that convert glucose to fatty acids. In turn, fatty acid synthesis promotes tumor growth [21] and disrupts the balance between apoptosis and cell proliferation, promoting the development of colorectal cancer.

A third possible mechanism is based on overexpression of both PGC1α and PGC1β, which increases the electron transfer activity of the mitochondrial respiration chain and augments mitochondrial biogenesis [11, 14]. Both processes partly disrupt ΔΨm and cytosolic calcium [Ca2+] buffering ability, with two consequences: 1) Dysfunctional oxidative phosphorylation in mitochondria, and 2) stimulation of the Ca2+ signaling cascade, which in turn may activate genes involved in tumor invasion and metastasis. Both of these effects can upregulate PGC1α, generating a positive feedback loop [11]. In addition, the increase in mitochondrial biogenesis seems to give the cells the necessary energy for increased longevity and cellular division, promoting tumor growth [11, 14].

From these three molecular mechanism we can conclude that PGC1α and PGC1β allow cells to balance i) mitochondrial activity and cytotoxic protection in the production of reactive oxygen species, and ii) apoptosis and cell proliferation [10, 11, 14, 21]. In addition, we can observe that all studies in cell lines [10, 11, 21] and mice [14] support the idea that high expression of PGC1α carries increased CRC risk. Note that while none of these articles work with the same cell line, the cells used are similar since the majority of cell lines are epithelial (Table 2). Shin et al. [10] use human colon cancer cells (HT29 and SNU-C4) and mouse colon cancer cells (CT-26). Srivastava et al. [11] use VACO425 and VACO429, another type of human colon cancer cell. Bhalla et al. [21] use other human colon adenocarcinoma cells, Colo205, as well as HT29, like Shin et al. Despite this, all of these studies arrived at the same conclusions.

However, Ilenia D’Errico [24] show that Bax is necessary for the pro-apoptotic effect of PGC1α in colorectal cancer cells, which could be another molecular mechanism of action, although this is not consistent with other studies. In this study [24], PGC1α overexpression seems to induce Bax translocation to mitochondria, and Bax-protein mediates the pro-apoptotic effect of PGC1α [24]. In this case, the cell line used is HCT116 (human colon carcinoma, also epithelial cells) and they also use mice who were subcutaneously injected with these cells. Here there is some controversy: in absence of Bax, PGC1α overexpression was not able to oppose tumor growth.

In addition to this research on overexpression of PGC1a, other studies have focused on low expression, inhibition or silencing of PGC1α and PGC1β. Using the same culture cells as Ilenia D’Errico [24], as well as other cells and in inmunodeficient mice, Fisher et al. [23] showed that PGC1β is aberrantly expressed in human colon cell lines and tumors, and maintains ERRα levels, contributing to its tumorigenic properties. These authors also found that KSR1 (Kinase suppressor of Ras 1) and AMPK (AMP-activated protein kinase), which act upstream of the PGC1β promotor, were linked to the action of transcriptional regulators PGC1β/ERRα (estrogen-related receptor α). Reduced KSR1 and AMPK expression also downregulated expression of PGC1β and ERRα, which inhibits the survival of colorectal cancer cells [23].

Based on all of this evidence, it seems that PGC1α/PGC1β is overexpressed in human colon cell lines during tumor development, and this overexpression seems to be required for both cell proliferation and survival, and to reduce apoptosis [10, 11, 14, 21, 23]. Only one study obtained markedly different results despite using the same cell line as one of the above (HCT116), although this was based on Bax instead of PGC1α [24]. Note that while all of these studies were performed using different cell lines with genetic changes, most of these are human epithelial cells (Table 2). For this reason, different results could not be attributed to the use of different cell lines. We consider that we cannot explain the origin of this differences.

Our systematic search returned two studies that analyzed the effects of chemotherapy on PGC1α expression in cultured cells and colonosphere cultures (Table 2) [25, 26]. These studies suggest three other molecular mechanism of action of PGC1α. 1) Chemotherapy of colorectal tumor cells induces a SIRT1/PGC1a-dependent increase in oxidative phosphorylation that promotes tumor survival during treatment because chemotherapy induces a shift in tumor energy metabolism that protects tumor cells from cytotoxic damage [26]. Mechanistically, chemotherapy-induced DNA damage results in increased expression of SIRT1, which deacetylates and thereby activates PGC1α as a transcriptional coactivator. PGC1a acts in concert with several transcription factors to stimulate the expression of genes involved in mitochondrial biogenesis and respiration, resulting in increased oxidative phosphorylation and helping them survive treatment. In this case, the authors worked with human colonosphere cultures [26]. 2) PGC1α-induced activation of Uridine phosphorylase (UPase) expression, which is mediated by an estrogen related receptor (ERR) binding site. Overexpression of PGC1α via this mechanism sensitizes colon cancer cells to growth inhibition by 5-deoxy-5-fluorouridine, presumably by inducing apoptosis in tumor cells [25]. In this way, 3) ERRα is primarily thought to regulate energy homeostasis by interacting with PGC1α or PGC1β. ERRα overexpression reduces sensitivity to chemotherapy, while inhibition of these genes reduces reactive oxygen species and ΔΨm, which increases sensitivity to chemotherapy [22, 23]. Here, Fisher et al. worked with HCT116 and other cell lines [23], but studied the effect of the chemotherapy on HepG2 [22].

These mechanisms support the conclusion mentioned above, that PGC1α and PGC1β allow cells to balance i) mitochondrial activity and cytotoxic protection in the production of reactive oxygen species, and ii) apoptosis and cell proliferation. In addition, they suggest that any therapy that reduces PGC1α expression will increase cancer cells’ sensitivity to chemotherapy. Thus, more and more authors support the idea of using PGC1α as a potential target in cancer therapy [21–23, 25, 26]. In fact, Do et al. [28] reported a strategy to reduce PGC1α levels to improve cancer therapy, though not in colon cancer cells. They showed for the first time that metformin, an insulin-lowering agent [29], induces miR-34a which reduces Sirt1 in wild-type p53 cancer cells, but does not occur in altered p53 cell lines. This fact was shown in HCT116 and MCF-7 cell lines among others. However, they only could show in an MCF-7 cell line (Breast cancer cells) that the reduction of Sirt1 involved reduced PGC1α and, subsequently, reduced NRF2 and enhanced susceptibility to oxidative stress [28]. DeCensi et al. 2010 [29] showed using a meta-analysis that metformin is associated with decreased risk of cancer, including colon cancer, in diabetic patients compared with other treatments [29].

Our systematic search returned three articles based on human samples. First, Ussakli et al. [6] studied 9 non-dysplastic colon biopsies from Ulcerative Colitis (UC) patients with high-grade dysplasia or cancer and 9 dysplasia-free UC patients, and concluded that while the development of dysplasia is preceded by mitochondrial loss, mitochondria are restored in cancer cells, which suggests that they are needed for further proliferation. This bimodal pattern may be driven by transcriptional regulation of mitochondrial biogenesis by PGC1α [6], which is consistent with the results of the cell culture studies described above [11, 14].

Two other papers propose the same idea, that overexpression of PGC1α results in higher CRC risk, although these studies are not comparable with the one described above [6]. First, Gaustadnes et al. [27] screened a selection of SNPs in pooled DNA, and found that the rs96516 SNP in the PPARGC-1A (*604517) 3’UTR was significantly associated with sporadic CRC risk, although this result was not reproducible in the same study [27]**.** Second, Feilchenfeldt et al. [13] studied expression levels of all isoforms of PPAR-ϒ and transcriptional partners such as PGC1α in patients with different stages of colon cancer, and found that expression levels of PPAR-ϒ vary between isoforms and cancer stages, while those of PGC1 were reduced in all cancer samples, with respect to normal samples [13].

Summarizing, while D’Errico et al., 2011 [20] showed that PGC1α^−/−^ mice are susceptible to intestinal tumorigenesis, several papers addressing the role of PGC-1 in tumor cell lines showed a role of PGC-1 in tumor progression. Thus, the function of PGC1α in colorectal cancer risk is not entirely clear, although it seems likely that it has a role in this disease.

## Conclusions

Colorectal cancer is a major global public health problem and the second leading cause of cancer-related death. Our systematic review indicates that altered expression of PGC1α modifies CRC risk. Most studies showed that overexpression of this gene increases CRC risk [10, 11, 14, 21, 23, 25, 26], while some studies indicated that lower than normal expression levels could increase CRC risk [6, 13]. Thus, various authors suggest that PGC1α is a good candidate as a molecular target for cancer therapy. Reducing expression of this gene could help to reduce risk or progression of CRC [22, 25, 26]**,** at least in different cell lines and transgenic or nude mice. However, in our opinion, there are not enough data on the role of PGC1α using human tumor samples to conclude a role of this gene in CRC. This question should be investigated further.

## Additional file

Additional file 1: Table S1. Reasons for exclusion of 22 articles according to abstract and full-text. Table that describes the reasons for exclusion of the selected articles in the first review. **Table S2.** Full details of the key characteristics of the selected studies. Table that describes the details of the selected articles. (PDF 245 kb)

## Abbreviations

ACBP: Acyl-CoA-binding protein
AMPK: AMP-activated protein kinase
CRC: Colorectal cancer
ERRa: Estrogen-related receptor α
Fig.: Figure
KSR1: Kinase suppressor of Ras 1
PGC-1: Peroxisome proliferator-activated receptor gamma coactivator 1
PPARGC1A or PGC1α: Peroxisome proliferator-activated receptor gamma coactivator 1-α
Sp1: Specificity protein 1
UC: Ulcerative Colitis
UPase: Uridine phosphorylase
ΔΨm: Mitochondrial membrane potential

## References

[1] Ferlay J, Shin HR, Bray F, Forman D, Mathers C, Parkin DM (2010). Estimates of worldwide burden of cancer in 2008: GLOBOCAN 2008. Int J Cancer.

[2] Vega P, Valentín F (2015). Cubiella. J Colorectal cancer diagnosis: Pitfalls and opportunities World J Gastrointest Oncol.

[3] Abraha I, Giovannini G, Serraino D, Fusco M, Montedori A (2016). Validity of breast, lung and colorectal cancer diagnoses in administrative databases: a systematic review protocol. BMJ Open.

[4] Smolková K, Plecitá-Hlavatá L, Bellance N, Benard G, Rossignol R, Ježek P (2011). Waves of gene regulation suppress and then restore oxidative phosphorylation in cancer cells. Int J Biochem Cell Biol.

[5] Bianchi G, Martella R, Ravera S, Marini C, Capitanio S, Orengo A (2015). Fasting induces anti-Warburg effect that increases respiration but reduces ATP-synthesis to promote apoptosis in colon cancer models. Oncotarget.

[6] Ussakli CH, Ebaee A, Binkley J, Brentnall TA, Emond MJ, Rabinovitch PS (2013). Mitochondria and tumor progression in ulcerative colitis. J Natl Cancer Inst.

[7] Jose C, Bellance N, Rossignol R (2011). Choosing between glycolysis and oxidative phosphorylation: a tumor’s dilemma?. Biochim Biophys Acta Bioenerg.

[8] Jones AWE, Yao Z, Vicencio JM, Karkucinska-Wieckowska A, Szabadkai G (2012). PGC-1 family coactivators and cell fate: roles in cancer, neurodegeneration, cardiovascular disease and retrograde mitochondria-nucleus signalling. Mitochondrion.

[9] Mazzanti R, Giulivi C (2006). Coordination of nuclear- and mitochondrial-DNA encoded proteins in cancer and normal colon tissues. Biochim Biophys Acta Bioenerg.

[10] Shin SW, Yun SH, Park ES, Jeong JS, Kwak JY, Park JI (2015). Overexpression of PGC-1α enhances cell proliferation and tumorigenesis of HEK293 cells through the upregulation of Sp1 and acyl-CoA binding protein. Int J Oncol.

[11] Srivastava S, Barrett JN, Moraes CT (2007). PGC-1α/β upregulation is associated with improved oxidative phosphorylation in cells harboring nonsense mtDNA mutations. Hum Mol Genet.

[12] Villena JA (2015). New insights into PGC-1 coactivators: redefining their role in the regulation of mitochondrial function and beyond. FEBS J.

[13] Feilchenfeldt J, Bründler MA, Soravia C, Tötsch M, Meier CA (2004). Peroxisome proliferator-activated receptors (PPARs) and associated transcription factors in colon cancer: reduced expression of PPARγ-coactivator 1 (PGC-1). Cancer Lett.

[14] Bellafante E (2014). PGC-1β promotes enterocyte lifespan and tumorigenesis in the intestine. Proc Natl Acad Sci U S A.

[15] Seale P (2015). Transcriptional regulatory circuits controlling brown fat development and activation. Diabetes.

[16] López-Lluch G, Irusta PM, Navas P, De Cabo R (2008). Mitochondrial biogenesis and healthy aging. Exp Gerontol.

[17] Van Gisbergen MW, Voets AM, Starmans MHW, De Cood IFM, Yadakd R, Hoffmanne RF (2015). How do changes in the mtDNA and mitochondrial dysfunction influence cancer and cancer therapy? Challenges, opportunities and models. Mutat res. Rev Mutat Res.

[18] Popay J, Roberts H, Sowden A, Petticrew M, Arai L, Rodgers M (1995). Guidance on the conduct of narrative synthesis in systematic reviews. Biostats.

[19] Moher D, Liberati A, Tetzlaff J, Altman DG, PRISMA GROUP. Preferred Reporting (2009). Items for systematic reviews and meta-analyses: the PRISMA statement. Ann Intern Med.

[20] D’Errico I, Salvatore L, Murzilli S, Lo Sasso G, Latorrec D, Martelli N (2011). Peroxisome proliferator-activated receptor-gamma coactivator 1-alpha (PGC1alpha) is a metabolic regulator of intestinal epithelial cell fate. Proc Natl Acad Sci U S A.

[21] Bhalla K, Hwang BJ, Dewi RE, Ou L, Twaddel W, Fang H (2011). PGC1α promotes tumor growth by inducing gene expression programs supporting lipogenesis. Cancer Res.

[22] Wu F, Wang J, Wang Y, Kwok TT, Kong SK, Wong C (2009). Estrogen-related receptor α (ERRα) inverse agonist XCT-790 induces cell death in chemotherapeutic resistant cancer cells. Chem Biol Interact.

[23] Fisher KW, Das B, Seok Kim H, Clymer BK, Gehring D, Smith DR (2015). AMPK Promotes Aberrant PGC1β Expression to Support Human Colon Tumor Cell Survival.

[24] D’Errico I, Lo Sasso G, Salvatore L, Murzilli S, Martelli N, Cristofaro M (2011). Bax is necessary for PGC1α pro-apoptotic effect in colorectal cancer cells. Cell Cycle.

[25] Kong X, Fan H, Liu X, Wang R, Liang J, Gupta N (2009). Peroxisome proliferator-activated receptor gamma Coactivator-1 alpha enhances Antiproliferative activity of 5Ј-Deoxy-5-Fluorouridine in cancer cells through induction of uridine phosphorylase. Mol Pharmacol.

[26] Vellinga TT, Borovski T, de Boer VCJ, Fatrai S, Van Schelven S, Trumpi K (2015). SIRT1/PGC1a-dependent increase in oxidative phosphorylation supports chemotherapy resistance of colon cancer. Clin Cancer Res.

[27] Gaustadnes M, Ørntoft TF, Jensen JL, Torring N (2006). Validation of the use of DNA pools and primer extension in association studies of sporadic colorectal cancer for selection of candidate SNPs. Hum Mutat.

[28] Do MT, Kim HG, Choi JH, Jeong HG (2014). Metformin induces microRNA-34a to downregulate the Sirt1/Pgc-1α/Nrf2 pathway, leading to increased susceptibility of wild-type p53 cancer cells to oxidative stress and therapeutic agents. Free Radic Biol Med.

[29] DeCensi A, Puntoni M, Goodwin P, Cazzaniga M, Gennari A, Bonanni B, Gandini S (2010). Metformin and cancer risk in diabetic patients: a systematic review and meta-analysis. Cancer Prev Res.

