# Supplementary material for: Alterations in PGC1α expression levels are involved in colorectal cancer risk: a qualitative systematic review
Source: BMC Cancer. 2017 Nov 9;17:731. doi: 10.1186/s12885-017-3725-3 (PMC5679491; doi:10.1186/s12885-017-3725-3)
Supplement: Additional file 1: Table S1. — Reasons for exclusion of 22 articles according to abstract and full-text. Table that describes the reasons for exclusion of the selected articles in the first review. Table S2. Full details of the key characteristics of the selected studies. Table that describes the details of the selected articles. (PDF 245 kb) [file 12885_2017_3725_MOESM1_ESM.pdf]

Mechanisms of action of PGC1α in relation to colorectal cancer development: A qualitative systematic review.

Jéssica Alonso-Molero*[1,2], Carmen González-Donquiles[1], Tania Fernández-Villa[1], Fernanda de Souza-Teixeira[1,3], Laura Vilorio-Marqués[1], Antonio J. Molina[1]. Senior Author: Vicente Martín[1,4]

Supplementary material:

Table S1: Reasons for exclusion of 22 articles according to abstract and full-text

Title	Excluded by abstract
WNT11 expression is induced by estrogen-related receptor alpha and beta-catenin and acts in an autocrine manner to increase cancer cell migration.	The article mentions ERRα, which is related to PGC1α, but it does not deal with PGC1α itself.
Estrogen receptor-related receptor alpha (ERRalpha) and oestrogen receptors (ERalpha and ERbeta) exhibit different gene expression in human colorectal tumour progression.	The article mentions ERRα, which is related to PGC1α, but it does not deal with PGC1α itself.
Rapid and cost effective genotyping method for polymorphisms in PPARG, PPARGC1 and TCF7L2 genes	This article does not deal with the colorectal cancer.
NF-kappa B/PPAR gamma and/or AP-1/PPAR gamma 'on/off' switches and induction of CBP in colon adenocarcinomas: correlation with COX-2 expression	The article mentions PPARS, but does not deal with the gene of interest.
CLA and n-3 PUFA differentially modulate clinical activity and colonic PPAR-responsive gene expression in a pig model of experimental IBD	The article mentions PPARS, but does not deal with the gene of interest.
IFN-ϒ and TNF-α treated mesenchymal stem cells can enhance the chemotherapy resistance of colon cancer cells	The article mentions Bax and Bcl-2, which are related to PGC1α, but does not deal with the gene of interest.
Impact of age, BMI and HbA1c levels on the genome-wide DNA methylation and mRNA expression patterns in human adipose tissue and identification of epigenetic biomarkers in blood	The article mentions various genes related to PGC1α, but does not deal with the gene of interest.
A marker-derived gene network reveals the regulatory role of PPARGC1A, HNF4G, and FOXP3 in intramuscular fat deposition of beef cattle	This article does not deal with the colorectal cancer.
A Six Months Exercise Intervention Influences the Genome-wide DNA Methylation Pattern in Human Adipose Tissue	This article does not deal with the colorectal cancer.

Coordinate changes in histone modifications, mRNA levels, and metabolite profiles in clonal INS-1 832/13 β-cells accompany functional adaptations to lipotoxicity	This article does not deal with the colorectal cancer.
Genetic mapping of quantitative trait loci for meat quality and muscle metabolic traits in cattle	This article does not deal with the colorectal cancer.
Epigenetic silencing of peroxisome proliferator- activated receptor ϒ is a biomarker for colorectal cancer progression and adverse patients' outcome	The article mentions PPARS, but does not deal with the gene of interest.
A PGC-1α-O-GlcNAc transferase complex regulates FoxO transcription factor activity in response to glucose	This article does not deal with the colorectal cancer.
The functional genetic variant Arg324Gly of frizzled-related protein is associated with colorectal cancer risk	Arg324Gly is not a PGC1α SNP.
Kinase Suppressor of Ras 1 (KSR1) Regulates PGC1α and Estrogen-Related Receptor α To Promote Oncogenic Ras-Dependent Anchorage-Independent Growth	This article does not deal with the colorectal cancer.
Involvement of mitochondria in apoptosis of cancer cells induced by photodynamic therapy	This article does not deal with the colorectal cancer or PGC1α.
Metformin induces microRNA-34a to downregulate the Sirt1/Pgc-1 alpha/Nrf2 pathway, leading to increased susceptibility of wild-type p53 cancer cells to oxidative stress and therapeutic agents	This article does not deal with the colorectal cancer or PGC1α.
Polymorphisms in fatty acid metabolism-related genes are associated with colorectal cancer risk	This article does not deal with PGC1α.
Polyphenolic profile and antiproliferative activity of bioaccessible fractions of zinc-fortified fruit beverages in human colon cancer cell lines	This article does not deal with PGC1α.

Title	Excluded by full-text
Estrogen-related receptor α (ERRα) inverse agonist XCT-790 induces cell death in chemotherapeutic resistant cancer cells	The article mentions PGC1α and its relationship with cancer, but does not deal with colorectal cancer.
Dietary supplementation of krill oil attenuates inflammation and oxidative stress in experimental ulcerative colitis in rats.	The article mentions PGC1α and its relationship with ulcerative colitis, but does not deal with colorectal cancer.
Simulated colon fiber metabolome regulates genes involved in cell cycle, apoptosis, and energy metabolism in human colon cancer	The article mentions PGC1α and colorectal cancer but not define a relationship between them. It is more focused on PPARα and other

cells.	genes.

Table S2: Full details of the key characteristics of the selected studies

Title	First Author	Year	Study population	CRC and other diseases	Demographics factors	Genes/Proteins	Reactive Oxygen Species (ROS) assessment	Primary results	Secundary results	Conclusion
**Overexpression of PGC1-alpha enhances cell proliferation and tumorigenesis of HEK293 cells through the upregulation of Sp1 and Acyl-CoA binding protein.**	Sung-Won Shin	2014	Cells Lines: HEK293, HT29 y SNU-Ca, CT26	CRC	Cells were subcutaneosly injected into the bilateral flanks of 6- to 7-week-old female immunodeficient mice. En ellas se inyectaron las células transfectadas anteriores.	PGC1-α; ABCP; Sp1	Yes	1) PGC-1α accelerates proliferation of HEK293 and CT-26 cells. 2)Knockdown of PGC1-α expression results in decreased cell proliferation of human colorectal cancer cells. 3)PGC-1α promotes the oncogenic potential of HEK293 cells. 4) PGC-1α-overexpressing HEK293 cells has a decreased sensitivity to oxidative stress.	5) Overexpression of PGC-1α may lead to upregulation of ACBP in HEK293 cells. 6) Downregulation of ACBP leads to decreased cell proliferation and increased sensitivity to H_2O_2-induced apoptosis. 7)PGC-1α does not physically interact with ACBP. The promoter of ACBP has an Sp1 binding site that is used to PGC1-α to increase ACBP expression.	PGC-1α overexpression upregulates proliferation of HEK293 and CT26 cells. In adition, this expression correlates with enhanced tumorigesis. Moreover, PGC-1α siRNA transfection resulted in decreased cell proliferation. Further studies to clarify the molecular interactions are needed.
**PGC-1β promotes enterocyte lifespan andtumorigenesis in the intestine**	Elena Bellafante	2014	Mice overexpressing PGC-1β and PGC-1β knockout mice	CRC	This study were carried out in male mice	PGC-1β, who is highly similar to PGC-1α.	Yes	1) PGC-1β Is Highly Expressed in the Intestinal Epithelium and Modulates Intestinal Morphology. 2) Intestinal PGC-1β Overexpression Enhances Antioxidant Defense. 3) Intestinal PGC-1β Overexpression Promotes Intestinal Carcinogenesis.	4) PGC-1β overexpression in the intestine is able to induce mitochondrial functions and respiration. 5) Intestinal PGC-1β Ablation Decreases Antioxidant Defense and Intestinal Carcinogenesis.	Thus, PGC-1β seems to act as an adaptive self-point regulator, capable of providing a balance between mitochondrial activity and increased ROS production.
**Mitochondria and Tumor Progression in Ulcerative Colitis**	Cigdem Himmetoglu Ussakli	2013	First patients set: 9 UC nonprogressors to CRC + 9 UC progressors to CRC + Five non-UC colon as normal controls; Second patients set: 6 UC progressors with different stages of dysplasia.	Ulcerative colitis and CRC	20 to 50 years old **Controls** (Mean: 35 years), 3 men y 2 women 20 to 77 years old **UC nonprogressor** (Mean: 48.8 years), 6 men y 3 women. 35 to 70 years old **UC progressor** (Mean: 52 years), 7 men y 2 women. 48 to 58 years old **UC progressors 2** (Mean: 42.8 years), 4 men y 2 women.	COX and PGC1-α. Telomeres length and mitochondrial DNA.	Not	1) Comparison of COX in Nondysplastic Biopsies of UC Progressors and Nonprogressors show that the tumor development could be due to a previous lower COX levels. 2) Cox levels increase after the tumor development (Bimodal pattern). Mitochondrias follow the same pattern. 3) PGC1α could be the driver of the mitochondrial changes observed.	4) Mitochondria and telomeres follow the same U-shaped pattern in tumor progression. 5)Telomeres lenght are indirectly PGC1α-regulated. (This association is not clear)	1) At the biomarker level, COX loss precedes tumor progression in UC. 2) At the biological level, the loss of COX represents a reduction of the number of mitochondria in preneoplasia, which is restored in cancers. It appears to be driven by PGC1α.
**PGC1α promotes tumor growth by inducing gene expression programs supporting lipogenesis.**	Kavita Bhalla	2011	PGC1α -/- and PGC1α +/+ mice; Colo205 and HT29 cell line. Colo205 were subcutaneously injected to mouse.	CRC	Colon carcinogenesis was induced by injecting mice once per week with 10mg/kg AOM for 8 weeks. Mice were monitored for 25 weeks and then euthanized.	PGC1α; PGC1β; ERRα; TAG	Not	1) Loss of PGC1 protects against both colon and liver tumorigenesis. 2) Overexpression PGC1α promotes tumor growth *in vivo*. 3)PGC1α mediated induction of fatty acid synthesis promotes tumor growth.	4) PGC1α promotes the expression of genes driving de novo fatty acid synthesis. Although ERRα is not responsible for the effects of PGC1α on lipogenic gene expression. 5) PGC1α promotes lipogenesis.	1) Novel role for PGC1α in promoting carcinogenesis and tumor growth. 2) PGC1α coordinates the induction of a gene expression program that facilitates the conversion of glucose to fatty acids. 3) PGC1α represents a potential therapeutic target for chemoprevention.
**Bax is necessary for PGC1α pro-apoptotic effect in colorectal cancer cells**	Ilenia D'Errico	2011	Bax+/- and Bax-/- HCT116 cells. This cells were subcutaneously injected to the subscapular region of an athymic mouse.	CRC	Nude mice randomly divided into two groups: Negative control and treatment group.	Bax; PGC1α	Not	1)PGC1α induces Bax activation. 2) PGC1α increases mitochondrial activity. 3) PGC1α induces apoptosis in presence of Bax, but not without Bax. 4)PGC1α inhibits tumor growth in presence of Bax.	5) In absence of Bax, PGC1α overexpression is no more able to oppose tumor growth.	1) In the presence of Bax, the PGC1α-induced ROS accumulation represents one of the main apoptosis-driving factors in CRC cells. 2) PGC1α is able to induce Bax activation and translocation to mitochondria, thus leading apoptotic cascade.
**PGC-1α/β upregulation is associated with improved oxidative phosphorylation in cells harboring nonsense mtDNA mutations**	Sarika Srivastava	2007	Cells lines: VACO 425 y VACO V429	CRC	VACO 425 and VACO V429 were grown in minimal essential medium supplemented with nonessential amino acids, glutamine, insulin, transferrin, hydrocortisone, sodium selenite, pyruvate, uridine and fetal bovine serum (FBS).	COXI; ND5; PGC1α; PGC1β; GFP	Not	1) PGC-1α and PGC-1β are markedly upregulated in V425. 2) Overexpression of PGC-1α and PGC-1β transcriptional coactivators stimulates mitochondrial respiration, at least in osteosarcoma cybrid. 3) Overexpression of PGC-1α stimulates complex IV activity. 4) Overexpression of PGC-1α/β transcriptional coactivators can stimulate respiration in OXPHOS deficient cells,	5) Mitochondrial DNA mutations in V425 do not abolish endogenous cell respiration despite affecting complex I and IV activities. 6) However, this mutations abolish respiration when transferred to a different nuclear background (e.g. osteosacorma). 7) Rate of respiration and complex IV activity are highly sensitive to KCN inhibition in V425. 8) Mitochondrial membrane potential and calcium buffering capacity are lowered in V425.	1)In V425 cells, the Ca^{2+}-dependent signaling events are active for relatively longer periods, which in turn might activate the nuclear genes (including PGC-1α/β) involved in tumor invasion and metastasis. 2) Overexpression of PGC-1α/β can stimulate respiration in OXPHOS deficient cells. 3) This pathway could be explored as a therapeutic approach for the treatment of human mitochondrial diseases.
**Validation of the Use of DNA Pools and PrimerExtension in Association Studies of SporadicColorectal Cancer for Selection of Candidate SNPs**	Mette Gaustadnes	2006	Two pools of genomic DNA: 1) 230 patients with diagnosed sporadic CRC and 2) 540 controls	Sporadic CRC	**Case pool**: mean age = 57.3 years; 40% female-60% male; Caucasians. **Control pool**: mean age= 62.6 years; 40% female - 60% male; Caucasians.	SNPs involved in the wnt/b-catenin pathway. SNPs in other candidate genes likely to be involved in CRC (e.g. PGC1α)	Not	Results were analyzed by the χ^2 test with a level of significance α= 0.05. Five SNPs were found. The SNP analysis of the (*604517)3'utr96516 was not reproducible, but it was always statistically significant.	1) A lot of other SNPs, not interesting in this review. 2) The method based on pooled samples of DNA is a valuable screening tool for identifying candidate SNPs associated with a given phenotype.	The results of this article allow concluded that the difference between cases and controls would be statistically significant for n=600 cases and n=600 controls.

Title	First Author	Year	Study population	CRC and other diseases (e.g. Ulcerative colitis o Chron´s disease)	Demographics factors	Genes/Proteins	Reactive Oxygen Species (ROS) assessment	Primary results	Secundary results	Conclusion
**SIRT1/PGC1a-Dependent Increase in Oxidative Phosphorylation Supports Chemotherapy Resistance of Colon Cancer**	Thomas T.Vellinga	2015	Cell culture were obtained from human colorectal tumours or liver reception.	CRC and liver metastasies. Chemotherapy resistance.	Human colorectal tumor specimens were obtained from patients undergoing colon or liver resection for primary or metastatic adenocarcinoma. And 12-week-old male mice who were injected subcutaneously transfected cells.	Electron transport chain complex; Mitochondrial ribosomal proteins; SIRT1; PGC1α	Not	1) Chemotherapy induces SIRT1 to promote oxidative energy metabolism. This gene controls mitochondrial biogenesis by deacetylating and activating PGC1α. 2) SIRT1 and PGC1α protect colon cancer cells against chemotherapy.	3)Chemotherapy of colorectal liver metastases induces changes in gene expression. 4) Genes regulating mitochondrial biogenesis and OXPHOS are upregulated in chemotherapy-exposed liver metastases. 5) Chemotherapy treatment induces oxidative energy metabolism.	Colorectal tumors shift their energy metabolism when challenged with chemotherapy. Chemotherapy induces OXPHOS in colon cancer cells via the SIRT1/PGC1a axis to help them survive treatment.
**AMPK Promotes Aberrant PGC1β Expression To Support Human Colon Tumor Cell Survival**	Kurt W. Fisher	2015	Seven different cell lines. The most used HCT116 and HCECs as control.	CRC	*In vivo* and *in vitro* culture cells.	PGC1α; PGC1β; ERRα; KSR1; K-Ras; AMPK	Not	PGC1α is not detected in HCT116 cell line. 1) PGC1β and ERRα are key downstream effectors of K-Ras, KSR1, and AMPK1. 2) Both AMPK 1 and K-Ras depletion decreased the protein levels of PGC1β. 3)PGC1β and ERRα are overexpressed in colon cancer and are required for colon cancer survival both *in vivo* and *in vitro*.	4) KSR1 regulates anchorage-independent growth and tumor maintenance. 5) AMPK seems to be a functional analog of KSR1. 6) An isoform ofAMPK promotes colorectal cancer cell survival.	The aberrant expression of PGC1β and ERRα persists in additional tumors with oncogenic Ras alleles will reveal the importance of these transcriptional regulators in the creation of tumor cells and the promotion of their survival. This fact could be a new therapeutic target.
**Peroxisome proliferator-activated receptor-γcoactivator 1-α (PGC1α) is a metabolic regulator of intestinal epithelial cell fate**	Ilenia D'Errico	2011	HT29, C2C12 and 143B cell line. iPGC1α/$Apc^{Min/+}$ mice	CRC	iPGC1α transgenic mice; iPGC1α $Apc^{Min/+}$ mice; C57BL/6-$Apc^{Min/+}$ mice.	PGC1α	Yes	1) Expression level of PGC1α in the intestine is higher in differentiated enterocytes than in the proliferative compartment at the bottom of the crypts, where it has only a scattered expression. 2) PGC1α Induces Mitochondrial Proliferation and Activation in Human Intestinal Cancer Cells. 3)PGC1α Induces Tissue-Specific ROS Accumulation and Apoptosis. 4) PGC1α Stimulates Intestinal Mitochondrial Biogenesis and Respiration *in vivo*, and suppresses Colorectal Carcinogenesis	5) PGC1α Proapoptotic Effect Is Lost in HT29ρ0 Cells. 6) A synthetic SOD2 and Catalase Mimetic EUK134 Abolishes the PGC1α Proapoptotic Effect. 8) PGC1α−/− Mice Are Susceptible to Intestinal Tumorigenesis.	1) PGC1α expression levels could influence intestinal epithelial cell fate by inducing mitochondrial-related metabolic modifications that induce apoptosis. 2) PGC1α overexpression is able to stimulate mitochondrial biogenesis, metabolic activities and accumulation of ROS. 3) In tissues with high aerobic energy demand, PGC1α is able to preserve ROS homeostasis; In normal intestine, PGC1α is not able to induce the ROS scavenging systems.
**Peroxisome Proliferator-Activated Receptor ? Coactivator-1alpha Enhances Antiproliferative Activity of 5'-Deoxy-5-Fluorouridinein Cancer Cells through Induction of Uridine Phosphorylase**	Xingxing Kong	2009	Cell lines: Colo320, HTC116, HepG2 y SK-BR-3.	Chemotherapy in CRC and Breast cancer.	Breast cancer and colon cancer cell lines	PGC1α; UPase	Not	1) PGC-1 Induces the Expression of UPase in Breast and Colon Cancer Cells. 2)PGC1α-Dependent Induction of UPase Gene in Cancer Cells Is Mediated by ERRα. 3) Overexpression of PGC-1 Sensitizes Cancer Cells to 5 -DFUR.	4) PGC-1 -Induced UPase Transcription Is Mediated by Binding of a Nuclear Receptor.	1) PGC-1α seems to be a regulator of UPase gene transcription, whose effect is mediated by ERRα . 2) PGC1α has an effect in absence of ERRα,suggesting the involvement of other regulatory factors. 3) In tumor cells, UPase catalyzes the transformation of 5 -DFUR to 5-FU, which inhibits their proliferation. In this way, PGC1α enhances the cell sensitivity to the treatment.
**Peroxisome proliferator-activated receptors (PPARs) and associated transcription factors in colon cancer: reducedexpression of PPARg-coactivator 1 (PGC-1)**	Jonas Feilchenfeldt	2004	17 patients with colon cancer	CRC in differents Dukes stage.	17 patients: Eleven patients were women (65%) and six patients were men (35%). Median age were 65 years (range 40–80 years). Twelve patients (71%) had tumors of Dukes stage A or B and only five patients (29%) had Dukes stage C.	PPARs; PGC1; RXRα	Not	1) RXRα expression in tumours is similar relative to normal mucosa. 2) PGC-1 expression in the tumors was significantly decreased relative to normal mucosa.	1)Expression levels of PPAR were correlated to Dukes stage.	1) PPARβ/δ may act as repressor of PPARα and PPARY target gene expression. 2) Reduced coactivator levels of PGC-1 are compatible with a reduced transcriptional activity of PPARY and hence a reduced tumor suppressor activity. 3)Transcriptional activity of PPARY may not only be decreased by mutation and the increase of the transcriptional repressor PPARβ/δ but also by downregulation of the coactivator PGC-1 of PPARY.
